# Validation of the What Matters Index: A brief, patient-reported index that guides care for chronic conditions and can substitute for computer-generated risk models

**DOI:** 10.1371/journal.pone.0192475

**Published:** 2018-02-22

**Authors:** John H. Wasson, Lynn Ho, Laura Soloway, L. Gordon Moore

**Affiliations:** 1 Centers for Health and Aging, Dartmouth Medical School, Lebanon, NH, United States of America; 2 North Kingstown Family Practice, North Kingstown, RI, United States of America; 3 3M Health Information Systems, Silver Spring, MD, United States of America; Yokohama City University, JAPAN

## Abstract

**Introduction:**

Current health care delivery relies on complex, computer-generated risk models constructed from insurance claims and medical record data. However, these models produce inaccurate predictions of risk levels for individual patients, do not explicitly guide care, and undermine health management investments in many patients at lesser risk. Therefore, this study prospectively validates a concise patient-reported risk assessment that addresses these inadequacies of computer-generated risk models.

**Methods:**

Five measures with well-documented impacts on the use of health services are summed to create a “What Matters Index.” These measures are: 1) insufficient confidence to self-manage health problems, 2) pain, 3) bothersome emotions, 4) polypharmacy, and 5) adverse medication effects. We compare the sensitivity and predictive values of this index with two representative risk models in a population of 8619 Medicaid recipients.

**Results:**

The patient-reported “What Matters Index” and the conventional risk models are found to exhibit similar sensitivities and predictive values for subsequent hospital or emergency room use. The “What Matters Index” is also reliable: akin to its performance during development, for patients with index scores of 1, 2, and ≥3, the odds ratios (with 95% confidence intervals) for subsequent hospitalization within 1 year, relative to patients with a score of 0, are 1.3 (1.1–1.6), 2.0 (1.6–2.4), and 3.4 (2.9–4.0), respectively; for emergency room use, the corresponding odds ratios are 1.3 (1.1–1.4), 1.9 (1.6–2.1), and 2.9 (2.6–3.3). Similar findings were replicated among smaller populations of 1061 mostly older patients from nine private practices and 4428 Medicaid patients without chronic conditions.

**Summary:**

In contrast to complex computer-generated risk models, the brief patient-reported “What Matters Index” immediately and unambiguously identifies fundamental, remediable needs for each patient and more sensibly directs the delivery of services to patient categories based on their risk for subsequent costly care.

## Introduction

The increasing prevalence of non-communicable, chronic disease is a major global health problem. The dominant strategy applied to control the escalating cost of chronic disease management is based on computer-generated risk models (CRMs) constructed from insurance claims and medical record data that designate a few patients at greatest risk for requiring costly care; these patients become targets for intensive interventions. Unfortunately, considerable evidence has exposed the substantial limitations of the CRM strategy [1–7].

Three deficiencies render CRM-based interventions inherently ill-advised. First, CRMs cannot make accurate predictions for individual patients [8]. For example, a minority of the highest-risk decile use the hospital within two years, in contrast to almost three times as many patients not designated high-risk who nonetheless require hospital resources [9,10]. In practice, this large false positive rate wastes scarce resources on the many patients in the highest-risk subgroup who will not use costly care, while care is relatively rationed for those not designated as at-risk, including the many false negatives destined to use costly services. From a public policy perspective, CRM-based targeting may perpetuate underinvestment in chronic disease prevention and management [11].

Second, CRMs based on demographics, diagnoses, and past use do not provide specific, real-time guidance for needs that matter to patients. Rather, CRMs output a general, asynchronous designation of risk, offered with the implicit assumption that clinicians can select and apply corrective action that will mitigate that risk. This generality supports neither clinicians nor patients, who must struggle during a time-constrained visit to identify a few current concerns that might respond to a management plan and thus decrease risk.

Third, CRMs are based on “what is the matter” (such as diagnoses and test results), rather than “what matters” to patients (such as bothersome symptoms, specific functional limits, and their quality of life). Thus, CRMs are often too abstract, untimely, or irrelevant to support patient engagement in care, and patient engagement in care is increasingly recognized as a highly effective strategy for delivering health care in the face of rising demand and shrinking budgets [12].

The authors of this research report recently tested the hypothesis that a clinical prediction rule based on a few self-reported measures may address the inadequacies of current CRM-based interventions for patients with chronic conditions [13]. We named this clinical prediction rule the “What Matters Index” (WMI) because it proved to be an appropriate indicator of patients’ quality of life—that is, what matters to patients. The WMI is based on a concise set of memorable measures that can be addressed by immediate actions and a management plan, and for which there is significant evidence that action can positively impact patient outcomes [14–21]. The proposed index is evaluated by summing the five binary scores, with an index of 0 representing a patient with the fewest reported problems and an index of 5 representing a patient with the most reported problems. The five WMI measures are listed in Table 1.

**Table 1:** Patient-reported measures in the “What Matters Index” (WMI).

Patient-Reported Measure
**Insufficient Health Confidence** How confident are you that you can manage and control most of your health problems? *(Not very confident or somewhat confident*, *scored as 1; versus very confident*, *scored as zero)*
**Pain** During the past four weeks, how much bodily pain have you generally had? *(Extreme or moderate pain*, *scored as 1; versus none*, *very mild*, *or mild*, *scored as zero)*
**Emotions** During the past four weeks, how much have you been bothered by emotional problems such as feeling anxious, irritable, depressed, or sad? *(Extremely or quite a bit*, *scored as 1; versus not at all*, *a little*, *or somewhat*, *scored as zero)*
**Polypharmacy** How many prescription medicines are you taking more than three days a week? *(More than five*, *scored as 1; versus 5 or less*, *scored as zero)*
**Adverse Effects from Medicines** Do you think any of your pills are making you sick? *(Yes or maybe*, *scored as 1; versus no*, *scored as zero)*

In addition to its foundation in clinical evidence for likely impact on patient outcomes, the WMI proved to be strongly associated with a history of emergency and hospital use when retrospectively tested in three populations: ages 18–64 (n = 8619), 50–64 (n = 7408), and 65+ (n = 3566). For example, regardless of a patient’s financial status, a WMI ≥ 2 was associated with approximately twice the odds of costly health care usage compared to a WMI = 0; for WMI ≥ 3, usage was approximately three times higher than for WMI = 0. The WMI’s positive predictive value was found to be comparable to a CRM based on multiple diagnoses and medications [13]. These preliminary results suggested that the WMI can adequately stratify risk levels (relative to a CRM) and immediately guide care that matters to patients. However, retrospective results guarantee neither future performance nor applicability in practice. Therefore, this report prospectively compares the WMI to two representative CRMs and illustrates how the WMI can be used to promote health care provider and patient engagement in improving health care delivery and health outcomes.

## Materials and methods

### Participants, data sources, and outcomes

Patient members and office practices of a Midwestern statewide Medicaid program were asked to complete a comprehensive, free, online health assessment called HowsYourHealth (www.HowsYourHealth.org) [22]. The branching logic of the online assessment includes the five WMI items, in addition to queries regarding demographics, symptoms, concerns, function, conditions, experience of care, preventive interventions, and past use of services. Of the 26,130 adults who completed the survey in 2014, 8771 fulfilled the eligibility criteria for this prospective assessment, which were identical to those used to develop the WMI and were based on patient self-identification of at least one of five chronic conditions—hypertension, cardiovascular disease, diabetes, respiratory disease, or arthritis—or use of at least one chronic medication. Subsequent emergency and hospital utilization information based on insurance claims data was available for all patients; however, the claims data indicated only the occurrence of emergency or hospital use, not frequency of use. Of the 8771 eligible Medicaid patients with one year of outcome data, 152 had incompletely responded to the WMI variables and were eliminated from the analysis.

### Predictors

The predictors listed in Table 1 are identical to those used to develop the WMI. We selected five binary (yes or no, 1 or 0) measures from a previous distillation of patient-reported “vital signs” [23]. By design, these measures are immediately available from patients, without requiring data retrieval from electronic health records or insurance claims; easily interpretable and translatable; and limited in number so that they are more easily memorized [24]. The sum of the five measures, a number from 0 to 5, constitutes the WMI—a direct expression of what matters to patients.

First, insufficient health confidence is an easy-to-measure representation of a patient’s lack of ability to manage health problems. A low level of self-management capacity predicts poor engagement in self-care and is associated with increased use of costly health care services [14–17]. The second and third predictors—emotional problems and pain—significantly impact the attainment of health confidence over time [18]. These measures are fundamental to the human condition and considerably influence health and use of services. Furthermore, emotional problems and pain often respond to simple behavioral interventions and are frequently assessed as vital signs in clinical settings [19,20]. The final two predictors, polypharmacy and medication side effects, account for a large percentage of preventable hospital and emergency department uses [21]. Multiple medications can cause harmful interactions, and even without such interactions, perceived side effects can reduce adherence [15].

### Representative CRMs

To evaluate the advantages or disadvantages of the WMI, we compared it to two representative CRMs commonly employed to assess risks for patients with chronic conditions. First, the Centers for Medicare and Medicaid Services in the United States suggest the use of a CRM to select patients for complex care reimbursement. In our study, to simulate the Medicare CRM requirement, we considered patients complex and at-risk when they reported both that they are taking three or more medications and that they had two or more chronic conditions. Second, a proprietary CRM, the 3M^TM^ Clinical Risk Groups, uses insurance claims to assign individuals to one of a set of risk groups based on historical clinical and demographic characteristics; these risk groups can be combined to predict costly care [25].

### Analysis

#### Predictive reliability of the WMI

The number of patients in the study population who were expected to require emergency or hospital care easily surpassed the minimum of five to ten observations per measure predicted emergency or hospital uses) that has been suggested for the development and validation of clinical prediction rules [26,27]. To test the association between the WMI sums and emergency or hospital use during the year after the patient self-assessments, odds ratios compared the likelihood that patients with higher WMI sums would use emergency or hospital care versus patients with a WMI of 0.

We do not know the characteristics of patients who were not solicited or who were either unable or unmotivated to complete the assessment. Therefore, we used logistic regression to examine if the WMI’s capacity to predict emergency or hospital use might have been vitiated by variations in the respondents’ self-reported characteristics of age, gender, number of chronic conditions (listed above), and poverty (i.e., sometimes or always not able to pay for essentials such as food, clothing, or housing). To supplement this validation, we also examined the WMI’s replicability in two very different patient groups: 1061 mostly older patients from nine private practices and 4428 Medicaid patients without chronic conditions. Tables A and B in S1 File provide a detailed comparison of these supplemental analyses with the primary analysis focused on Medicaid patients with chronic conditions.

#### Comparison of the WMI to representative CRMs

In the Medicaid patients with chronic conditions, we compared the WMI and representative CRMs in three respects. First, we compared the models’ sensitivities and positive predictive values for costly care. Sensitivity is defined as the proportion of patients who actually used costly care who were correctly designated as at-risk by the model. Positive predictive value is defined as the proportion of patients designated as at-risk who actually became users of costly care. Because predictive values are influenced by prevalence, we adjusted the WMI or CRM test cut-points so that the comparisons would be based on similar proportions of “at-risk” patients.

Second, we analyzed the distribution of the five WMI measures among patients designated by the CRMs to be at higher or lower risk. Third, we examined the relationship between true positives and false positives using the area under the receiver operation characteristic curve (AUROC) expressed as the concordance statistic (c-statistic), which is frequently used to compare CRMs. The c-statistic approximates the overall accuracy of a binary classifier as its discrimination cut-point is varied: the c-statistic of a perfect classifier is 1.0, and a c-statistic of 0.50 indicates that the classification is no better than chance.

## Results

### Patient characteristics

Despite this Medicaid population’s youth (40% aged 18–49 and none over 65), it has a high prevalence of serious chronic conditions such as diabetes (31%), respiratory diseases (39%), and atherosclerosis (17%), and more than a third (35%) report taking more than 5 prescription medications. Most (70%) are sometimes unable to pay for food, clothing, and housing. More than 40% report that they lack confidence that they can manage and control most of their health problems. Additional characteristics of this population are described in Table A in S1 File.

### Predictive reliability of the WMI

**Fig 1:**
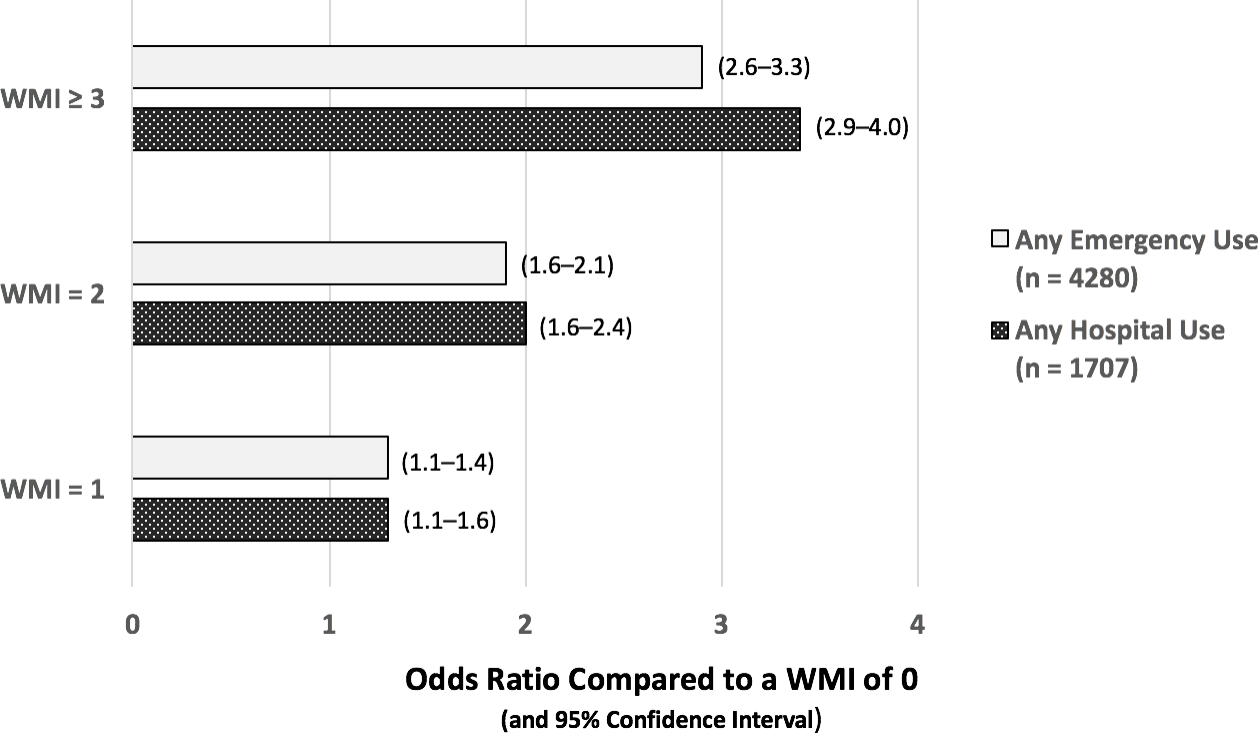
Odds ratios for subsequent use of costly care comparing patients with WMI > 0 to those with WMI = 0. Sample population: 8619 Medicaid patients; 95% confidence intervals.

During the year following their completion of the WMI assessment, half of the patients used the emergency department and 20% were admitted to a hospital. There was a strong association between WMI magnitude and increased use of hospital or emergency services during the subsequent year (Fig 1). The odds ratios (with 95% confidence intervals) for subsequent hospitalization of patients with WMI sums of 1, 2, and ≥ 3 were 1.3 (1.1–1.6), 2.0 (1.6–2.4), and 3.4 (2.9–4.0); for emergency room use, the corresponding ratios were 1.3 (1.1–1.4), 1.9 (1.6–2.1), and 2.9 (2.6–3.3). These findings validate the pattern observed during the development of the WMI [13].

Logistic regression models considering age, gender, number of chronic conditions, and poverty indicated that, among these variables, the WMI was the one most highly associated with subsequent emergency or hospital use (p < 0.001).

### Comparison of WMI to representative CRMs

In Table 2, the proportions of patients designated at-risk by the WMI and by each CRM have been matched so that their sensitivities and predictive values can be compared. The Medicare CRM identifies roughly half the population as being at-risk, and to approximate the Medicare CRM target population, the WMI cut-point was set to ≥ 2. For comparably sized populations, the WMI and Medicare CRM sensitivities and positive predictive values for future hospital use were essentially the same. The predictive performances of the proprietary CRM and the WMI were also equivalent for comparably sized at-risk populations, implemented by setting the WMI cut-point for higher risk to ≥ 3. The overall accuracies (c-statistics) of the proprietary CRM and the WMI were the same (0.63). (The c-statistic cannot be calculated for Medicare CRM because its cut-point is fixed.)

**Table 2:** Sensitivities and predictive values for subsequent hospital use of the WMI and CRMs.

Method	WMI ≥ 2	Medicare CRM	WMI ≥ 3	Proprietary CRM
**Proportion of all patients designated “at-risk”**	0.53	0.52	0.30	0.30
**Sensitivity of method for emergency use**	0.62	0.56	0.38	0.35
**Sensitivity of method for hospital use**	0.69	0.64	0.45	0.43
Positive predictive value^*^ **for emergency use**	0.58	0.54	0.63	0.58
Positive predictive value^*^ **for hospital use**	0.26	0.25	0.30	0.28

* Positive Predictive Value: The proportion of patients designated by the method as “at-risk for emergency or hospital use” who actually used such care in the year following the assessment.

**Fig 2:**
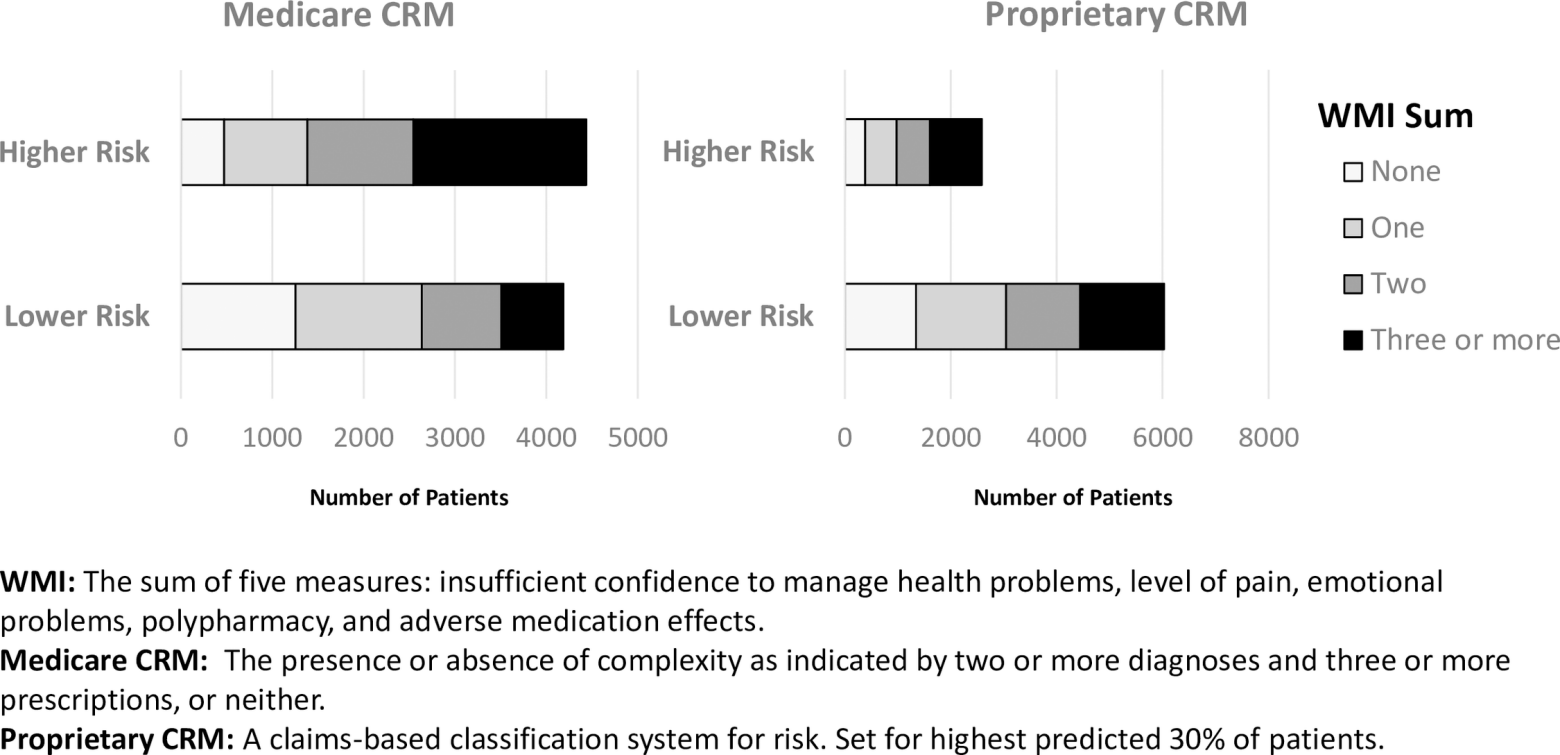
Distribution of WMI measures for Medicaid patients in relation to CRM risk levels.

Although either a CRM or the WMI can provide actuarial stratification to identify future risks for costly care, resource allocation based on only these forecasts is inefficient because of their low positive predictive values. However, Fig 2 shows that needs identified by the WMI are distributed among all patients and are not confined to the higher risk patients designated by the CRMs. For example, the proprietary CRM designated 984 and 1586 patients reporting a WMI score ≥ 3 as being in the higher and lower risk groups for hospital use, respectively. Thus, using CRMs to target resources ignores a large proportion of patients at risk for requiring future costly care. Moreover, CRMs are indifferent to potentially remediable risk factors that are easily identifiable from patient self-reports.

When a CRM based on clinical and laboratory data is combined with patient-reported data, an increase in the c-statistic is often documented [9,10,28]. However, in practice, incorrect classification persists even with such hybrid models, and the c-statistic gain is offset by the considerable effort required to combine the clinical, laboratory, and patient-reported inputs such that the output can be made available in a manner that is timely and useful for clinical practice. For example, when a hybrid risk model is established by combining a WMI ≥ 3 with the proprietary CRM’s highest risk decile, 290 (67%) and 186 (43%) of the 431 patients thus classified as the highest risk subgroup subsequently used emergency or hospital services, respectively. These predictive values represent only small improvement over those of the CRM highest risk-decile alone (59% and 37%, respectively).

## Discussion

### Implications

Despite extensive evidence to the contrary, the idea that population health and associated costs can be effectively managed with CRM-based interventions is entrenched in current practice. This report prospectively demonstrates the superior performance of the WMI in relation to two representative CRMs in terms of providing easily interpretable personal guidance for each patient as well as a sensible basis to allocate resources for many patients. Moreover, the WMI is both reliable and comparable to these CRMs in its capacity to forecast risk for costly care.

The WMI offers the following additional advantages:

- It has no direct cost.
- It equitably assesses the remediable needs of all patients, not only a designated few.
- It is unambiguous and is therefore much less likely to produce high variances in interpretation compared to the list of patients generated by a CRM.
- It correlates strongly with overall quality of life and can therefore be used to monitor the impact of interventions designed to improve patients’ quality of life.
- It applies in any setting because it is patient-reported and does not require insurance claims, electronic medical records, or complicated scoring methods.
- By design, the WMI is consistent with the intent of Article 22 of the European Union’s General Data Protection Regulation that requires decision logic to be explicable [29].

Self-reported instruments have long been applied to populations of community-dwelling older adults at risk for hospitalization, functional decline, institutionalization, and death [30]. Recent studies also document the value of patient report for the management of cancer. [31] The WMI broadens the use of self-reporting beyond these narrow circumstances, emphasizing just a few items to identify remediable needs for all adults.

## An illustration: Applying the WMI in a clinical setting

Each WMI item is meant to elicit an action that meets each patient’s needs. A very common and remediable risk factor included in the WMI is a patient’s lack of confidence in their ability to manage most health concerns; this risk factor is associated with many adverse health experiences, including more frequent (and often avoidable) emergency or hospital care use, lost time from work, and medical harm [15,16]. Applying the WMI model, patients who say they are not confident that they can control and manage most of their health problems are then asked by medical assistants or the online health assessment (www.HowsYourHealth.org) to answer the query, “What would it take for you to be able to say that you are very confident that you can control most of your health problems during the next two months”? The patients’ verbatim responses are included in a summary report for the clinicians who provide their care. Examples of queries for the other WMI items are listed elsewhere [13].

To illustrate how the WMI identifies population needs in a clinical setting, we summarized the verbatim responses to the online assessment of 1915 adult patients from across the United States. These patients met the identical selection criteria that were used to select the Medicaid population sample. (Table A in S1 File) The verbatim responses, in which patients identified the health care interventions that they perceived would be most effective, could be generally classified into the following four categories.

1. *Professional help*. Patients most often request better medical information and education, such as clarification of their diagnoses, timely sharing of test results, and when possible, additional relief of symptoms. Examples: (a) “Help of a doctor who will actually listen and take my problems seriously without just pushing medication.” Michigan; WMI = 2. (b) “If I got an accurately diagnosis of my illness, and able to get a specific course of treatment I could control and manage my health problems.” Texas; WMI = 2.
2. *Personal change*. Patients acknowledge their need to improve time-management, motivation, and lifestyle. Examples: (a) “Staying focused on what is required to be healthier.” New Hampshire; WMI = 2. (b) “More time and attention to my diabetes.” North Carolina; WMI = 2.
3. *Non-professional support and guidance*. Patients request coaching or support in the workplace, home, and/or community; financial assistance may also be needed. Examples: (a) “Finances are stopping me from getting medical help. Co-pays for doctors and medications has taken most of my life savings.” Rhode Island; WMI = 2. (b) “Need some coaching.” Minnesota; WMI = 2.
4. *Non-response or uninterpretable response*.

**Fig 3:**
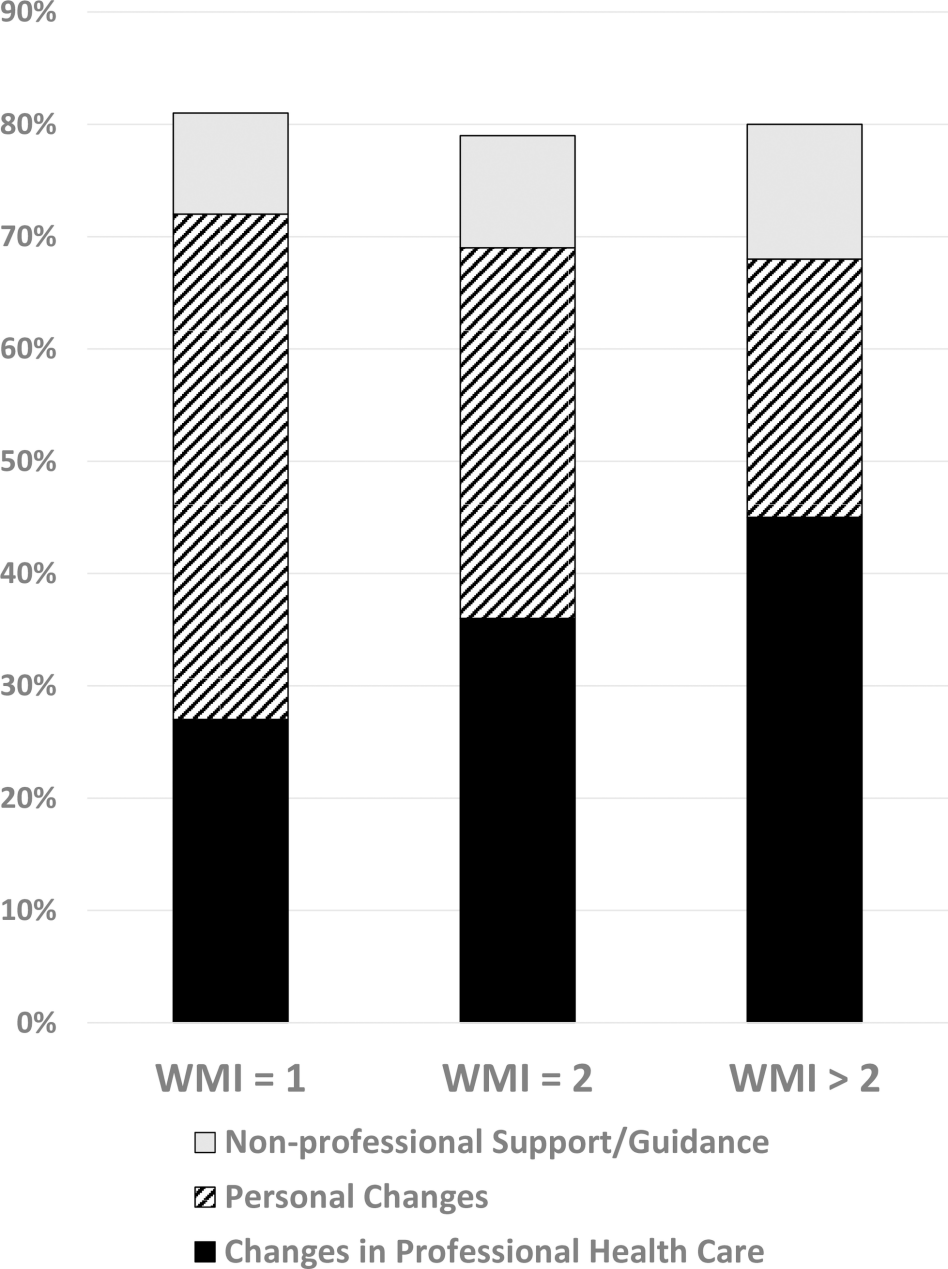
Influence of What Matters Index (WMI) on patient reports of changes needed to improve their health confidence.

Fig 3 compiles 1915 patients’ verbatim responses regarding changes they require to improve their health confidence, and illustrates how their needs vary in relation to their WMI sums.

For this sample population of adult patients with chronic conditions, higher WMI sums are strongly associated with an increased likelihood that the respondents identify a need for professional assistance, and with a reduced likelihood that they consider their personal behavior as the primary remediable cause for their low confidence. Logistic regression confirms the persistence of this pattern (p < 0.001) regardless of patient age, gender, financial status, or number of chronic conditions. For patients who used hospital or emergency care in the past year and had a WMI ≥ 3, half (70/142) believed that the event may have been avoidable; for those having a WMI = 1, approximately one in five (15/76) shared that belief.

In summary, people simply answer each question and bring their responses to the attention of someone who can help them address each problem, such as a health professional, a support group, a knowledgeable friend, or even a website like www.HowsYourHealth.org used to develop the WMI (22). Services appropriate for the level of risk based on “what matters” is the goal. Thus, this illustration demonstrates that to improve a patient’s low health confidence, more attention to medical diagnostics, therapeutics, and education is indicated when the WMI is high, whereas more support for behavioral change is indicated when the WMI is low. (An example of a WMI interface for public distribution is included in Table C in S1 File)

### Limitations

Several limitations of the WMI deserve comment. First, these prospective results were derived from a self-selected sample of Medicaid patients with chronic conditions who completed a health assessment; whether the WMI would perform similarly for different patients is a valid concern. To address this point, we examined the WMI in 1061 mostly older patients from nine private practices, selected using the same criteria for chronic conditions, as well as in 4428 patients with no chronic conditions from the Midwestern statewide Medicaid program. Among the private practice patients with chronic conditions, the odds ratios (with 95% confidence intervals compared to a WMI of 0) for subsequent emergency room use were 1.8 (1.1–2.8), 2.1 (1.2–3.6), and 3.0 (1.4–6.3) for patients with WMIs of 1, 2, or ≥3, respectively. For WMIs of 1 or ≥ 2, the odds ratios of subsequent hospitalization were 1.4 (0.8–2.6) and 2.4 (1.2–4.5), respectively. In the Medicaid population without chronic conditions, the odds ratios (with 95% confidence intervals compared to a WMI of 0) for subsequent emergency room use were 1.2 (1.03–1.40), 2.2 (1.73–2.76), and 3.2 (2.01–5.21) for patients with WMIs of 1, 2, or ≥3, respectively. For WMIs of 1 or ≥ 2, the odds ratios of subsequent hospitalization were 1.1 (0.87–1.48) and 1.6 (1.10–2.26), respectively. To summarize, the WMI’s prospective prediction of costly usage was replicable in three very different populations. Tables A and B in S1 File further detail the characteristics of all patients and the supplemental analysis procedures.

The WMI’s capacity to improve health outcomes and reduce costs is additionally limited by the extent to which CRMs are entrenched in health management practice. In other words, a critical sociological limitation of the WMI is, ironically, the challenge it represents to the flawed but widely adopted status quo. It is true that a small proportion of patients account for a large proportion of the costs of care; that CRMs can identify some patients who will cost more than others; and that payers can use computer algorithms to generate lists of these patients almost effortlessly and send them to medical practitioners who will, with incentives, act on the lists. However, evidence suggests that this approach is ineffective at controlling care costs, does nothing to specifically guide care for individual patients, and probably has negative consequences for those not targeted [1–7,13]. Similar inadequacies have been previously documented for intensive care management based on targeting distinct diseases, an antecedent to the current CRM-based interventions [32].

Finally, although a controlled cost-effectiveness trial has not yet been conducted to compare the value of the WMI and CRM-based strategies, and descriptions of the optimum intervention types and timing for the different WMI levels are not yet available, the WMI’s advantages strongly suggest that it is ethically more justifiable and economically more sensible to implement simple, self-reported measures to determine what matters to all patients and to use those results to guide care. Patient reporting is increasingly recognized as the most appropriate basis for chronic care management because of its ease of implementation and benefits for patients and the providers who serve them [22,33]. The WMI results validate the utility of parsimonious patient-reported measures in guiding the delivery of services that matter to patients [34].

## Conclusion

By considering what people say about their own health, the WMI identifies both important needs that matter and risks for costly health care use. In contrast to the complex CRM algorithms, which leave by far the greatest share of patients who use costly care in the low-risk category and do not provide standardized follow-up procedures for high-risk patients, the brief, unambiguous WMI can guide care plans that mitigate risks for all patients with chronic conditions and probably for people with no chronic conditions as well.

## Supporting information

S1 File (Text A) Further WMI verification in alternative population samples. (Table A) Additional Information for the Patient Populations Included in this Report. (Text B) Results of secondary validations. (Table B) Actual Uses Within the Subsequent Year Per 100 Patients in Each WMI-Based Risk Group. (Text C) Examples of a What Matters Index for Public Use. (Table C) Sample of a WMI Interface for Public Use.
(DOCX)
