# Supplementary material for: Validation of the What Matters Index: A brief, patient-reported index that guides care for chronic conditions and can substitute for computer-generated risk models
Source: PLoS One. 2018 Feb 22;13(2):e0192475. doi: 10.1371/journal.pone.0192475 (PMC5823367; doi:10.1371/journal.pone.0192475)
Supplement: S1 File — (Text A) Further WMI verification in alternative population samples. (Table A) Additional Information for the Patient Populations Included in this Report. (Text B) Results of secondary validations. (Table B) Actual Uses Within the Subsequent Year Per 100 Patients in Each WMI-Based Risk Group. (Text C) Examples of a What Matters Index for Public Use. (Table C) Sample of a WMI Interface for Public Use. (DOCX) [file pone.0192475.s001.docx]

**Supporting Information (S1)**

**Text A. Further WMI verification in alternative population samples.**

The primary WMI validation based on a Medicaid patient population with chronic conditions was further verified by prospective test results from two other populations: patients with chronic conditions from nine private practices and Medicaid patients without evidence of a chronic condition (no prescription medications and none of five chronic conditions—hypertension, cardiovascular disease, diabetes, respiratory disease, or arthritis). One year after the WMI assessment, 62 hospital uses and 117 emergency uses were observed for the private practice population, and 310 hospital uses and 1372 emergency uses were observed for the Medicaid population with no chronic conditions. Finally, open-ended responses were gathered from patients across the United States who completed the same online assessment (HowsYourHealth.org) as the Medicaid and private practice patients. The response period ranged from April 2015 to May 2017. This last group of patients all expressed a lack of confidence that they could manage and control most of their health problems, and they fulfilled the same selection criteria as the other patient groups: they were taking one or more chronic medications or had one of five chronic conditions (hypertension, cardiovascular disease, diabetes, respiratory disease, or arthritis). Table A details the characteristics of the different populations included in this report.

**Table A. Additional Information for the Patient Populations Included in this Report.**

|  | **Medicaid**  **Population for Primary Validation** | **Patients from Nine Private Practices for WMI Reliability Evaluation** | **Medicaid Patients Without Chronic Conditions for WMI Reliability Evaluation** | **U.S. Patients who Provided Illustrative Responses** |
| --- | --- | --- | --- | --- |
|  | ***n* = 8,619** | ***n* = 1,061** | ***n* = 4,428** | ***n* = 1,915** |
| **Demographics** | | | | |
| **Always able to buy essentials: food, clothing, housing** | **30%** | **94%** | **43%** | **72%** |
| **% female** | **58%** | **60%** | **55%** | **71%** |
| **Age 18-35** | **12%** | **4%** | **47%** | **28%** |
| **Age 36-49** | **28%** | **20%** | **27%** | **29%** |
| **Age 50-64** | **60%** | **50%** | **26%** | **32%** |
| **Age 65 and older** | **0%** | **26%** | **0%** | **11%** |
| **What Matters Index (WMI) Measures** | | | | |
| **Not health-confident** | **41%** | **37%** | **33%** | **100%** |
| **Moderate or extreme pain** | **45%** | **18%** | **10%** | **29%** |
| **Emotional problems** | **22%** | **7%** | **8%** | **25%** |
| **> 5 prescription medications** | **35%** | **9%** | **0** | **15%** |
| **Medications may be causing illness** | **17%** | **8%** | **0** | **12%** |
| **Other Measures of Illness Burden** | | | | |
| **Hypertension** | **70%** | **42%** | **0** | **35%** |
| **Atherosclerosis** | **17%** | **7%** | **0** | **7%** |
| **Diabetes** | **31%** | **9%** | **0** | **14%** |
| **Respiratory disease** | **39%** | **17%** | **0** | **19%** |
| **Arthritis** | **38%** | **31%** | **0** | **28%** |
| **Limited activities of daily living** | **23%** | **5%** | **3%** | **15%** |
| **Limited social support** | **15%** | **5%** | **10%** | **17%** |
| **Markers for Costly Care** | | | | |
| **Any hospital use in the past year** | **24%** | **7%** | **4%** | **14%** |
| **≥ 2 uses of hospital or emergency care** | **11%** | **3%** | **3%** | **10%** |
| **Prior use may have been unnecessary** | **19%** | **7%** | **1%** | **36%** |
| **Possible harm from health care in the past year** | **2%** | **1%** | **0%** | **4%** |
| **Utilization in the Year Following Completion of the WMI** | | | | |
| **Any emergency use** | **50%** | **11%** | **31%** | **NA** |
| **Any hospital use** | **20%** | **6%** | **7%** | **NA** |

**Text B. Results of secondary validations.**

Although the small number of hospital uses and the distribution of WMI responses did not support differentiating WMI = 2 from WMI ≥ 3 in the private practice patients, the overall pattern of the WMI as a predictor of future use is similar for the two populations with chronic conditions. For the private practice patients with chronic conditions, the odds ratios (with 95% confidence intervals compared to patients with a WMI of 0) for subsequent emergency room use were 1.8 (1.1–2.8), 2.1 (1.2–3.6), and 3.0 (1.4–6.3) for patients with WMIs of 1, 2, or ≥3, respectively. For WMIs of 1 or ≥ 2, the odds ratios of subsequent hospitalization were 1.4 (0.8–2.6) and 2.4 (1.2–4.5), respectively. In this private practice population, a WMI ≥ 2 approximated the proportion of private patients designated as at-risk by the Medicare CRM (0.17 for the WMI versus 0.19 for the CRM), and both methods produced similarly low positive predictive values for hospitalization (0.10 and 0.12, respectively). The proprietary CRM could not be applied to the private-practice patients.

In the Medicaid population without chronic conditions (as defined for this report), the association of a three-item WMI (absent queries about medications) with subsequent costly care was also maintained. The odds ratios (with 95% confidence intervals compared to a WMI of 0) for subsequent emergency room use were 1.2 (1.03–1.40), 2.2 (1.73–2.76), and 3.2 (2.01–5.21) for patients with WMIs of 1, 2, or ≥3, respectively. For WMIs of 1 or ≥ 2, the odds ratios for subsequent hospitalization were 1.1 (0.87–1.48) and 1.6 (1.10–2.26), respectively. Table B compares the prospective test results from the private practice patients and the Medicaid patients with no chronic conditions to the Medicaid population with which the primary prospective WMI test was validated.

**Table B.** **Actual Uses Within the Subsequent Year Per 100 Patients in each WMI-Based Risk Group^*^.**

|  | **Medicaid Patients with**  **Chronic Conditions** | | **Private Practice Patients with**  **Chronic Conditions** | | **Medicaid Patients with**  **NO Chronic Conditions** | |
| --- | --- | --- | --- | --- | --- | --- |
|  | **Hospital Use** | **Emergency Use** | **Hospital Use** | **Emergency Use** | **Hospital Use** | **Emergency Use** |
| **WMI = 0** | **11** | **37** | **4** | **8** | **6** | **28** |
| **WMI = 1** | **15** | **42** | **6** | **13** | **8** | **32** |
| **WMI ≥2** | **26** | **63** | **10** | **18** | **10** | **48** |

**^*^**Details on the numbers of patients with each WMI in each population are available in the data repository at: doi:10.5061/dryad.c50n5

**Text C. Examples of a What Matters Index for Public Use**

The What Matters Index can be used as part of a health assessment as for example <https://howsyourhealth.org> or in a paper based format as shown in Table C below.

**Table C. Sample of a What Matters Index Interface for Public Use.**

For Public Use. All other rights reserved. Copyright Trustees of Dartmouth College. 2017.

INSTRUCTIONS:

- Pick the one answer that describes you best for each of the five questions.
- Whenever your answer is in **BIG PRINT**, give yourself one point. You can have at most 5 points and at least, no points.
- Add up your points. People with a sum of two or higher have an increased risk of using the hospital or emergency room during the next year. Therefore, they need to make sure that doctors or nurses are aware of the needs and that they have good communication with all health professionals. They also benefit when they complete full check-up using [www.HowsYourHealth.org](http://www.HowsYourHealth.org) and bring the results (the Action Form) to their clinical appointments. HowsYourHealth also automatically creates a personal health record that can be used to keep track of their progress.
- A person with a sum of one should consider taking similar action as a person with a sum or two or more.
- If you are SOMEWHAT OR NOT VERY CONFIDENT, ask yourself “what would it take for you to be able to say that you are very confident that you can control most of your health problems during the next two months?” Write that plan here and share it with someone who can help you.

_____________________________________________________________________________________________________________
